# Dynamic Changes of Mitochondrial Fusion and Fission in Brain Injury after Cardiac Arrest in Rats

**DOI:** 10.1155/2017/1948070

**Published:** 2017-12-28

**Authors:** Yi Li, Qingqin Tang, Peng Wang, Jiahong Qin, Haidong Wu, Jiali Lin, Zitong Huang

**Affiliations:** ^1^Department of Emergency Medicine, The First Affiliated Hospital of Soochow University, Soochow, China; ^2^Institute of Cardiopulmonary Cerebral Resuscitation, Sun Yat-sen University, Guangzhou, China; ^3^Department of Clinical Laboratory Center, The First Affiliated Hospital of Soochow University, Soochow, China; ^4^Department of Emergency Medicine, Sun Yat-sen Memorial Hospital, Sun Yat-sen University, Guangzhou, China

## Abstract

Mitochondria change their morphology dynamically by continual fusion and fission processes to fulfill their function. However, little is known about the effect of cardiac arrest on mitochondrial dynamics. This study aimed to investigate time-dependent change of the mitochondrial dynamics after brain ischemic injury in rats of cardiac arrest. After resuscitation, obvious neuronal injury, reduced adenosine triphosphate (ATP) levels, excessive reactive oxygen species (ROS) generation, decreased mitochondrial membrane potential (MMP), and increased release of mitochondrial cytochrome c were observed at 12 h and 24 h after cardiac arrest. Moreover, we found that elongation of mitochondria was observed at 4 h after cardiac arrest, whereas fragmented mitochondria were significantly increased, along with concomitant increase in the fission proteins Drp1 and Fis1 and a reduction in the fusion proteins Mfn1 and Mfn2 at 12 h and 24 h after cardiac arrest. Taken together, these findings suggest that imbalance in mitochondrial dynamics probably contributes to brain injury after cardiac arrest.

## 1. Introduction

Global ischemic and hypoxic brain injury after cardiac arrest remains severe worldwide problem. Nearly 500,000 victims suffer cardiac arrest annually in North America with an overall mortality rate of 90% [1], and up to 80% of the survivors have poor neurological outcomes due to ischemic brain injury [2, 3]. Despite the obvious advancement in the field of cardiac arrest and resuscitation, global brain injury that involves a complex cascade of events remains a common cause of morbidity and mortality [4]. The brain relies primarily on oxidative phosphorylation for their function and is particularly sensitive to oxygen deprivation. This reliance has provided increasing focus on mitochondria for therapy after cardiac arrest.

Mitochondria are essential organelle which plays a key role to provide adenosine triphosphate (ATP) for brain function [5, 6], whereas detrimental alterations in mitochondrial function are a primary source of oxidative stress and induce neuronal cell apoptosis [7]. However, mitochondrial responses to ischemic brain injury induced by cardiac arrest are uncertain.

In the brain, mitochondria are dynamic organelles and exist in varying morphologies, ranging from small individual organelles to extended reticular networks. Mitochondrial morphology is altered in various disease conditions, including neurodegenerative diseases [8, 9], cardiovascular disease [10], diabetes [11], obesity [12], and cancer [13]. These changes in morphology are mediated by continuous fusion and fission process, which are important for maintenance of functional mitochondria. Mitochondrial fusion event is critically regulated by the proteins mitofusin 1 (Mfn1) and mitofusin 2 (Mfn2). On the other hand, dynamin-related protein 1 (Drp1) and fission 1 (Fis1) are main proteins that mediate mitochondrial fission in the brain [14]. Recently, we have demonstrated that Drp1 is associated with cardiac arrest and contributes to mitochondria-dependent apoptosis after cardiac arrest [15]. However, alternations between fusion and fission events with each other under conditions of cardiac arrest are not well understood.

The purpose of this study is to provide a descriptive evaluation of mitochondrial dynamics in response to ischemic brain injury after cardiac arrest. In this study, we applied a 6 min cardiac arrest rat model and characterized imbalance in mitochondrial fusion and fission in the brain after cardiac arrest.

## 2. Methods

### 2.1. Animal Preparation

Male Sprague-Dawley rats (*n* = 40, 10/group; body weight = 350–450 g) were purchased from the Laboratory Animal Center of Sun Yat-sen University. Animals were housed in light and temperature controlled environment. Food and water were supplied ad libitum. Studies were approved by the Institutional Animal Care and Use Committee of Sun Yat-sen University, and experiments were performed in accordance with the National Institutes of Health guidelines for the care and use of laboratory animals.

### 2.2. Experimental Protocol

Cardiac arrest was established by asphyxia and this procedure has been described in detail previously [16]. Briefly, asphyxia was induced by arterial injection of vecuronium (1 mg/kg) and clamp of the endotracheal tube. Approximately 4 min after asphyxia, cardiac arrest was determined by a mean arterial pressure (MAP) of ≤20 mmHg. After 6 min of cardiac arrest, rats were resuscitated with intravenous epinephrine (0.01 mg/kg), mechanical ventilation with 100% oxygen, and precordial compression. Resuscitation was stopped when there was restoration of spontaneous circulation (ROSC) or no ROSC after 4 min. ROSC was defined as the return of supraventricular rhythm with a MAP of ≥60 mmHg for a minimum of 5 min. One hour after ROSC, the rats were weaned from the ventilator and returned to the cage for the observation. After achieving ROSC, rats were randomized to 4 h, 12 h, and 24 h group by using random number table. And additional sham experiments were conducted with 100% oxygen, epinephrine, and same surgical procedures except cardiac arrest.

### 2.3. Neurological Function Examination

At 4 h, 12 h, and 24 h after ROSC, neurologic deficit scores were determined by two investigators blinded to the study groups as previously described [17]. In brief, scores are assessed by scoring 7 categories and ranges from 0 (brain death) to 80 (normal brain function). In this study, dead rats were excluded from the scores assessment.

### 2.4. ATP Assay

Adenosine triphosphate (ATP) levels in brain tissues were determined by the use of ATP assay kit (Beyotime, China). Briefly, brain tissues were washed and homogenized in ice-cold ATP-releasing buffer. Homogenates were centrifuged at 12,000*g* for 5 min and supernatants were separated for use immediately. Luminescence from a 100 *μ*L sample was assayed in a luminometer (Molecular Devices, SpectraMax M5) together with 100 *μ*L of ATP detection buffer and was then normalized by protein concentration (pmol ATP/mg protein).

### 2.5. ROS Production

Reactive oxygen species (ROS) production was measured using the tissue of ROS classical assay kit (Genmed, USA) that utilized 2,7-dichlorofluorescein diacetate (DCF-DA) as the oxidative fluorescent probe. After the fresh brain tissues were isolated, the levels of ROS were measured following the manufacturer's instructions within 1 h.

### 2.6. Mitochondrial Membrane Potential

Mitochondrial membrane potential (MMP) was measured using the fluorescent dye JC-1 (Beyotime, China) with buffer. The fluorescence of JC-1 at 490 nm (excitation wavelength) was observed by luminometer concurrently at 530 and 590 nm using plate reader (Molecular Devices, SpectraMax M5) with ratio of 590/530 which was used as relative value.

### 2.7. Cytochrome c Release

Mitochondrial and cytoplasmic extracts from brain tissues were prepared using mitochondrial protein extraction kit (Beyotime, China). The levels of cytochrome c in cytosolic and mitochondrial fractions were measured by the Quantikine rat cytochrome c immunoassay assay kit (R&D Systems, USA) according to the manufacturer's protocol. Data were expressed as ng/mg protein.

### 2.8. Transmission Electron Microscopy

The brain hemisphere was harvested and then selected cortex area was immediately immersed in cold 2.5% glutaraldehyde with 0.1 mol/L cacodylate buffer (pH 7.4), postfixed in 1% OsO_4_, dehydrated, and embedded in Epon. Serial ultrathin sections (60–80 *μ*m) were cut regardless of the orientation and mounted on copper grids and stained with lead citrate and uranyl acetate. Images were collected using a transmission electron microscope (FEI, Tecnai G2). Two blinded observers using Image J software analyzed mitochondrial images with similar approach as described previously [18].

### 2.9. Real-Time qRT-PCR

Total RNA was extracted from brain cortex using TRIzol solution (Invitrogen, USA) according to the manufacturer instructions. Reverse transcription was performed using PrimeScript RT master mix (TaKaRa, China). The specific primers for Mfn1, Mfn2, Drp1, Fis1, and *β*-actin (an internal control) were as follows: Mfn1, 5′-AGCTC AAGGTTGTGAGTCCT-3′ (forward), 5′-CATCCCCTGGGCTTTATTC A-3′ (reverse); Mfn2, 5′-AGCCTGGTGAGTGTGATGTG-3′ (forward), 5′-CTCCGTGGTGACATCGATCC-3′ (reverse); Drp1, 5′-CCAGGAATGACCAAGGTCCC-3′ (forward), 5′-CCTCGTCCATCAGGTCCAAC-3′ (reverse); Fis1, 5′-TTTGAATACGC-CTGGTGCCT-3′ (forward), 5′-TACCTTTGGGCAACAGCTCC-3′ (reverse); *β*-actin, 5′-CACGGCATTGTCACCAACTG-3′ (forward), 5′-AACACAGCCTGGATGGCTAC-3′ (reverse). RT-PCR was performed at 95°C for 2 min, followed by 40 cycles at 95°C for 3 s and at 60°C for 30 s. The relative mRNA expression levels of the target genes were measured using the 2^−ΔΔCt^ method normalized to mRNA levels for *β*-actin.

### 2.10. Western Blotting

Protein was extracted from cortex of each rat and placed in lysis buffer to measure global Mfn1, Mfn2, Drp1, Fis1, and *β*-actin expression. Protein samples (30 ug each) were separated on a 10% SDS-polyacrylamide gel and transferred to polyvinylidene difluoride membranes. After blocking the nonspecific binding sites with 5% nonfat milk for 60 min, membrane was incubated overnight at 4°C with antibodies including rabbit anti-Mfn1 (Cell Signaling Technology, 1 : 1000 dilution), rabbit anti-Mfn2 (Cell Signaling Technology, 1 : 1000 dilution), rabbit anti-Drp1 (Cell Signaling Technology, 1 : 1000 dilution), mouse anti-Fis1 (Santa Cruz, 1 : 500 dilution), and mouse anti-*β*-actin (Cell Signaling Technology, 1 : 5000 dilution). The membranes incubated with horseradish peroxidase-conjugated secondary antibody for 45 min at room temperature and developed using an enhanced chemiluminescence detection method (Cell Signaling Technology). The relative protein expression was quantified by using Quantity One Software (Bio-Rad, USA) and normalized to mRNA levels for *β*-actin.

### 2.11. Statistical Analysis

Data were expressed as mean ± standard deviation (SD). Statistical analysis was performed using SPSS version 20.0 (SPSS, Chicago, IL). Comparison of the same parameters among groups was done using one-way analysis of variance (ANOVA), and the difference between pairs of means was assessed post hoc with Tukey's test. Fisher's exact test was performed for neurologic deficit scores among groups. A value of *P* < 0.05 was considered statistically significant.

## 3. Results

### 3.1. Cardiac Arrest Induced Neurological and Mitochondrial Disorders

**Figure 1:**
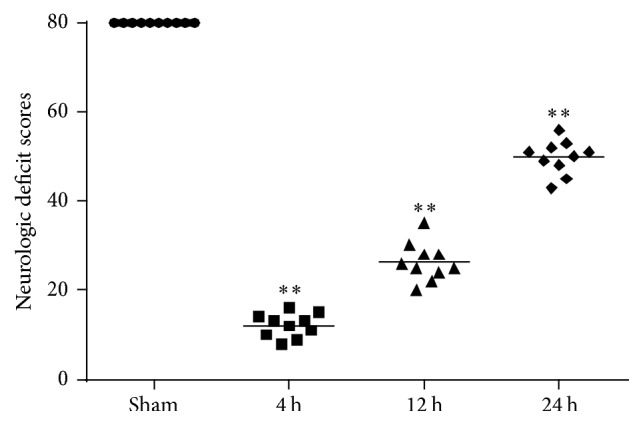
Neurologic deficit scores (NDS) were measured at 4 h, 12 h and 24 h after the restoration of spontaneous circulation (ROSC) in rats. The NDS ranged from 0 (brain death) to 80 (normal brain function). Horizontal bars present mean values and each dot represents one rat. ^*∗∗*^*P* < 0.01 versus sham.

**Figure 2:**
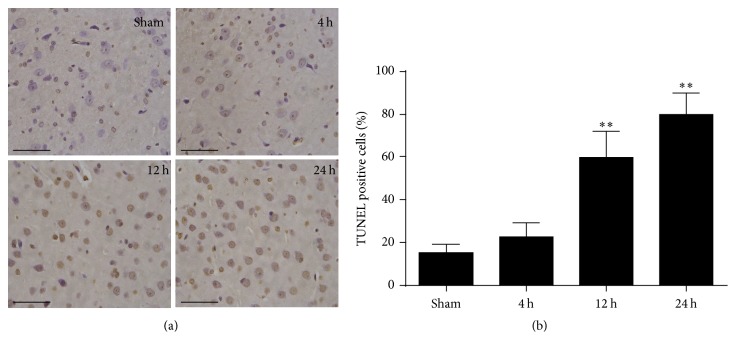
Representative images of TUNEL staining of the brain cortex after ROSC. (a) TUNEL staining in the brain cortex at 4 h, 12 h, and 24 h after ROSC. Scale bar = 10 *μ*m. (b) Quantification of the apoptotic neurons by positive staining. Data are represented as mean ± standard deviation (SD). *n* = 6 photomicrographs were counted per animal. ^*∗∗*^*P* < 0.01 versus sham.

To estimate neurological function performance after cardiac arrest, neurologic deficit scores were measured. A significant reduction of neurological scores was found in rats at 4 h, 12 h, and 24 h after ROSC (Figure 1). TUNEL staining was performed to determine the neuronal damage after cardiac arrest in rats. The apoptotic neuron gradually increased at 4 h and 12 h and peaked at 24 h after ROSC (Figure 2). Therefore, the neurologic deficit scores and apoptotic neuronal death indicate that neurological disorders occurred after cardiac arrest after ROSC.

**Figure 3:**
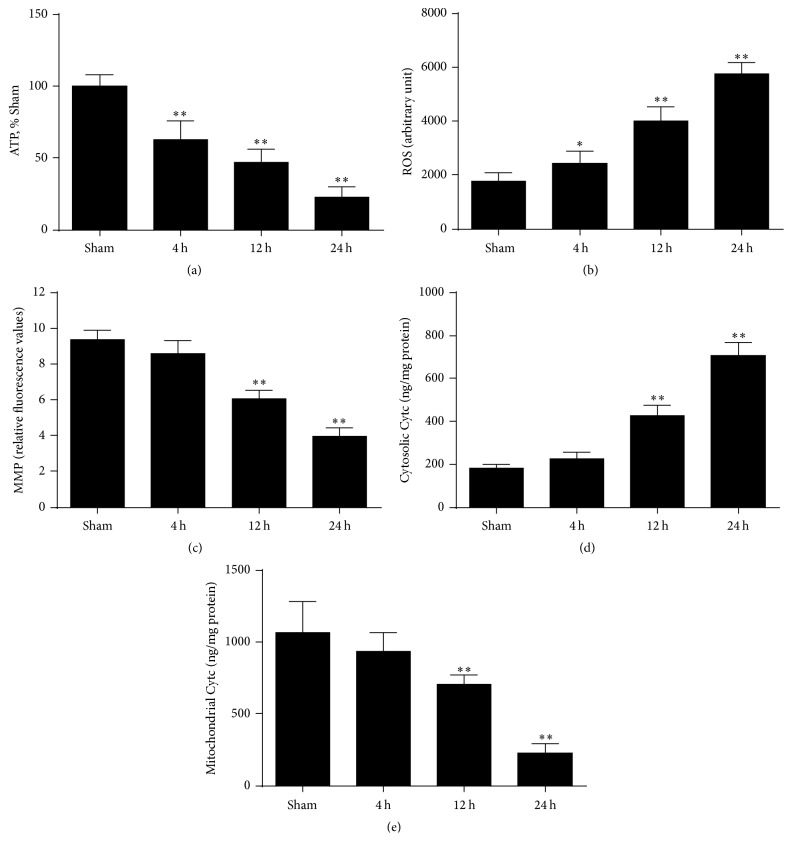
Mitochondrial function tests were measured in the ischemic brain at 4 h, 12 h, and 24 h after ROSC. (a) Brain adenosine triphosphate (ATP) levels. (b) Brain reactive oxygen species (ROS) production. (c) Brain mitochondrial membrane potential (MMP) levels. (d) Cytosolic cytochrome c and (e) mitochondrial cytochrome c levels. *n* = 6. Data are represented as mean ± SD. ^*∗*^*P* < 0.05, ^*∗∗*^*P* < 0.01 versus sham.

In addition to generating ATP, mitochondria are the major sources for ROS production and activation of proapoptosis factors. To investigate the effect of cardiac arrest on mitochondrial functional activity. We first examined ATP production in the brain, and the results revealed that brain ATP levels significantly decreased at 4 h, 12 h, and 24 h after ROSC (Figure 3(a)). As shown in Figure 3(b), we also measured the levels of ROS in the brain. The results showed that brain ROS generation progressively increased at 4 h, 12 h, and 24 h after ROSC. In addition, loss of MMP plays a key role in the apoptotic process for the release of certain apoptogenic factors and is therefore used to predict mitochondria-mediated apoptosis [19]. The results showed that brain MMP gradually decreased at 4 h, 12 h, and 24 h after ROSC (Figure 3(c)). But a gradual and significant increase of cytochrome c release from mitochondria was observed simultaneously (Figures 3(d) and 3(e)). Taken together, these data indicate that the impairment of mitochondrial function significantly increased with time after ROSC, especially at 24 h.

### 3.2. Cardiac Arrest Induced Dyshomeostasis of Mitochondrial Morphology

**Figure 4:**
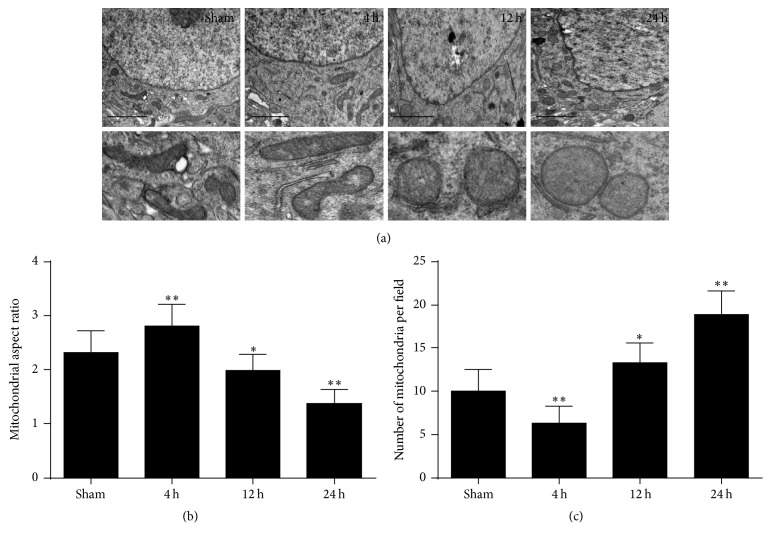
Representative electron microscopy (EM) images of mitochondrial morphology in the brain cortex after ROSC. (a) Electron micrograph of mitochondria in the brain cortex at 4 h, 12 h, and 24 h after ROSC. Scale bar = 1 *μ*m. (b) Mitochondrial aspect ratio was measured by the line tool and then the ranges were calculated and converted to their actual values using the scale bar. (c) Mitochondrial number was counted by two blinded observers and normalized to unit field. *n* > 300 mitochondria in each group. Data are represented as mean ± SD. ^*∗*^*P* < 0.05, ^*∗∗*^*P* < 0.01 versus sham.

To determine changes in mitochondrial morphology in the brain from the ischemic injury in the cardiac arrest model, electron microscopy was performed. As shown in Figure 4, global cerebral ischemia induced a dramatic mitochondrial fragmentation at 12 h and 24 h after cardiac arrest. Interestingly, by 4 h ROSC, we scarcely saw mitochondrial fragmentation but observed more elongated mitochondria. In the tissue sections of brain, the amounts and the shape of the mitochondria were performed by quantitative analysis to compare mitochondrial morphology more objectively. The mitochondrial number per field was significantly decreased at 4 h after ROSC and then steadily increased at 12 h and 24 h. The mitochondrial average aspect ratio underwent exactly an opposite alteration, with transiently increasing at 4 h after ROSC and decreasing gradually with time thereafter. Taken together, these results suggest that the alterations in mitochondrial morphology occurred from elongation to fragmentation with time after ROSC.

### 3.3. The Expression of the Mfn1 and Mfn2 Fusion Proteins Firstly Increased and Then Decreased in the Brain after Cardiac Arrest

**Figure 5:**
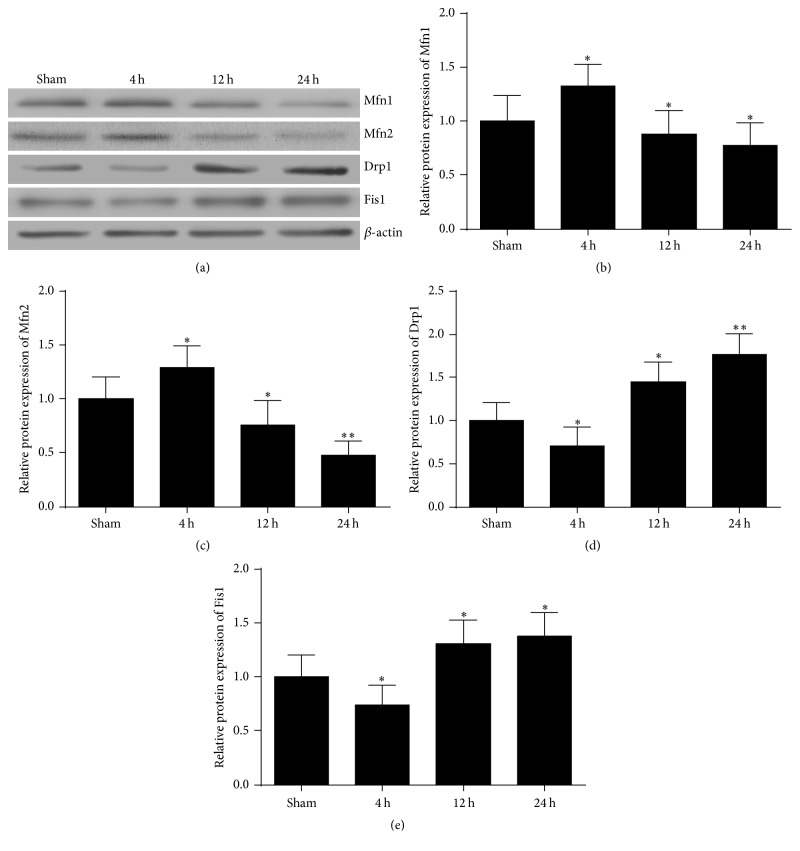
Effects of cardiac arrest on Mfn1, Mfn2, Drp1, and Fis1 protein expression in the brain cortex at 4 h, 12 h, and 24 h after ROSC. (a) Representative results of western blots analysis of Mfn1, Mfn2, Drp1, and Fis1 protein expression in the brain cortex. *β*-Actin was used as the internal control. (b) Quantitative analysis of Mfn1, (c) Mfn2, (d) Drp1, and (e) Fis1 protein expression in the brain at 4 h, 12 h, and 24 h after ROSC. *n* = 8. Data are represented as mean ± SD. ^*∗*^*P* < 0.05, ^*∗∗*^*P* < 0.01 versus sham.

**Figure 6:**
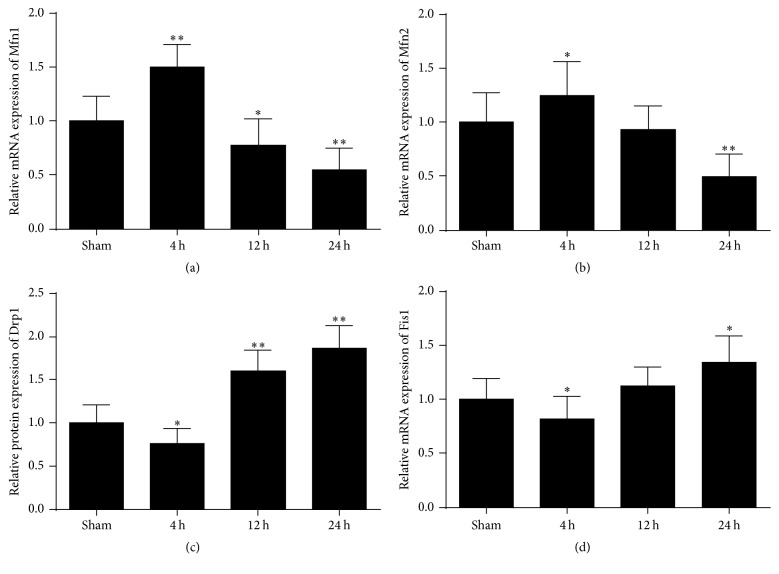
Effects of cardiac arrest on Mfn1, Mfn2, Drp1, and Fis1 mRNA levels in the brain cortex at 4 h, 12 h, and 24 h after ROSC. (a) Quantitative analysis of Mfn1, (b) Mfn2, (c) Drp1, and (d) Fis1 mRNA expression levels in the brain cortex at 4 h, 12 h, and 24 h after ROSC. *β*-Actin was used as the internal control. *n* = 8. Data are represented as mean ± SD. ^*∗*^*P* < 0.05, ^*∗∗*^*P* < 0.01 versus sham.

To examine if changes in mitochondrial morphology were associated with molecular mechanism of mitochondrial fusion and fission events after ROSC, we first investigated the time course of fusion proteins after cardiac arrest. Here, RT-PCR and western blot were performed to detect the mRNA and protein level of Mfn1 and Mfn2 at 4 h, 12 h, and 24 h after ROSC. As shown in Figures 5 and 6, the results revealed that both mRNA and protein levels of Mfn1 and Mfn2 significantly increased in the brain at 4 h after ROSC and then decreased significantly at 12 h and 24 h after ROSC. Therefore, these data show that cerebral mitochondrial fusion factor mRNA and proteins expression levels firstly increased at the early time point and then decreased with time after ischemic injury induced by cardiac arrest.

### 3.4. The Levels of the Drp1 and Fis1 Fission Proteins Firstly Decreased and Then Increased in the Brain after Cardiac Arrest

Next, we examined the effect of cardiac arrest on the levels of mitochondrial fission proteins. As shown in Figures 5 and 6, a remarkable reduction in Drp1 and Fis1 protein levels was observed at 4 h after ROSC, then increased at 12 h, and reached a peak at 24 h. Likewise, at 4 h, 12 h, and 24 h after ROSC, Drp1 and Fis1 mRNA levels also underwent an similar alteration with the protein levels. Taken together, these data suggest that cerebral mitochondrial fission factor mRNA and proteins expression levels firstly decreased and then increased at the later time after ischemic injury induced by cardiac arrest.

## 4. Discussion

In the present study, we found that an increase in elongated mitochondria at 4 h after cardiac arrest was accompanied by a concomitant increase in expression of the fusion proteins Mfn1 and Mfn2, along with a decrease in fission protein Drp1 and Fis1 levels, whereas opposite trend occurred at 12 h and 24 h after cardiac arrest, especially at 24 h. These results demonstrated that mitochondria became fusion temporarily at the early stage after cardiac arrest and a shift drove toward more fission subsequently and peaked at 24 h after cardiac arrest. Therefore, our results suggest that brain injury induced by cardiac arrest causes imbalance of fusion and fission.

Increasing evidences suggest excess mitochondrial fission facilitates apoptosis, whereas blocked fission events or enhanced mitochondrial fusion exhibit a protective effect. Previous studies have demonstrated that inhibition of mitochondrial fission reduces related mitochondrial apoptosis and improves myocardial and neurological outcomes after cardiac arrest [15, 20]. Mitochondrial fission can be repressed in response to protein kinase A- (PKA-) dependent Drp1 phosphorylation of Drp1 Ser637 due to increased cyclic adenosine monophosphate (cAMP) levels, resulting in elongation of the mitochondria with a higher density of cristae and a capacity for efficient ATP production [21, 22]. A recent report demonstrated that the A-kinase anchoring protein 1 (AKAP1) recruits PKA to the outer mitochondrial membrane to promote mitochondrial fusion reactions [23]. Thus, the AKAP1/PKA complex system may be involved in regulation of balance in mitochondrial fission and fusion and protection from brain injury after cardiac arrest.

The crucial importance of mitochondrial fusion and fission in the nervous system has been firmly established [24, 25]. The neuronal injury induced by mitochondrial fission has been exposed in various neurological diseases, such as Parkinson's disease, Alzheimer's disease, and Huntington's disease [26–28]. When fusion predominates over fission, mitochondria become elongate into a tubular interconnected network. In contrast, when the balance shifts toward fission, mitochondria initiate organelle division and result in short and round phenotype [29]. In the present study, the results showed that more stubby and swollen mitochondria were observed at 12 h and 24 h after cardiac arrest, coinciding well with neuronal injury and certain mitochondrial dysfunctions including decline in ATP levels, increase in ROS generation, reduction of MMP, and enhanced release of apoptotic factor cytochrome c at the same time points. Similar results have been reported in the previous study [20], but detection in the brain after cardiac arrest is novel. In this study, we failed to observe a dramatic change in the brain MMP and cytosolic and mitochondrial cytochrome c levels at 4 h after cardiac arrest. These delayed occurrences indicate that brain apoptotic process is relatively late due to cardiac arrest.

Our present study showed that the tendency of mitochondrial elongation and fusion was definitely apparent at 4 h after cardiac arrest, and the tendency of mitochondria fission existed at 12 h and 24 h after cardiac arrest. Evidence suggested that fusion was ceased by loss of the inner membrane potential [30]. Consequently we presume that fusion is impeded from 4 h after cardiac arrest due to MMP depolarization and soon initiate rapid fragmentation of mitochondria by fission inevitably. Furthermore, increased mitochondrial fusion may provoke quality control mechanism of mitochondria to maintain the neuron survival, which is consistent with unremarked neuronal damage at 4 h after cardiac arrest in the present study. Therefore, we believe that the tendency of mitochondrial fusion is primary adaptive responses to brain injury after the onset of cardiac arrest. However, the underlying mechanism has not been studied clearly.

## 5. Conclusion

In conclusion, our research shows that imbalance of the mitochondrial fusion and fission after cardiac arrest and resuscitation participates in ischemic brain injury. This study suggests mitochondrial fusion and fission proteins are potential key targets for brain injury after cardiac arrest.
